# Identification and characterization of a new sulfoacetaldehyde reductase from the human gut bacterium *Bifidobacterium kashiwanohense*

**DOI:** 10.1042/BSR20190715

**Published:** 2019-06-20

**Authors:** Yan Zhou, Yifeng Wei, Ankanahalli N. Nanjaraj Urs, Lianyun Lin, Tong Xu, Yiling Hu, Ee Lui Ang, Huimin Zhao, Zhiguang Yuchi, Yan Zhang

**Affiliations:** 1 Tianjin Key Laboratory for Modern Drug Delivery and High-Efficiency, Collaborative Innovation Center of Chemical Science and Engineering, School of Pharmaceutical Science and Technology, Tianjin University, Tianjin 300072, China; 2 Metabolic Engineering Research Laboratory, Institute of Chemical and Engineering Sciences, Agency for Science, Technology and Research (A*STAR), Singapore, Singapore; 3 Department of Chemical and Biomolecular Engineering, University of Illinois at Urbana-Champaign, 600 South Mathews Avenue, Urbana, IL 61801, U.S.A.

**Keywords:** NADH/NADPH, Nitrogen assimilation, Organosulfur degradation, Sulfoacetaldehyde reductase, X-ray crystallography

## Abstract

Hydroxyethylsulfonate (isethionate (Ise)) present in mammalian tissues is thought to be derived from aminoethylsulfonate (taurine), as a byproduct of taurine nitrogen assimilation by certain anaerobic bacteria inhabiting the taurine-rich mammalian gut. In previously studied pathways occurring in environmental bacteria, isethionate is generated by the enzyme sulfoacetaldehyde reductase IsfD, belonging to the short-chain dehydrogenase/reductase (SDR) family. An unrelated sulfoacetaldehyde reductase SarD, belonging to the metal-dependent alcohol dehydrogenase superfamily (M-ADH), was recently discovered in the human gut sulfite-reducing bacterium *Bilophila wadsworthia* (*Bw*SarD). Here we report the structural and biochemical characterization of a sulfoacetaldehyde reductase from the human gut fermenting bacterium *Bifidobacterium kashiwanohense* (*Bk*TauF). *Bk*TauF belongs to the M-ADH family, but is distantly related to *Bw*SarD (28% sequence identity). The crystal structures of *Bk*TauF in the apo form and in a binary complex with NAD^+^ were determined at 1.9 and 3.0 Å resolution, respectively. Mutagenesis studies were carried out to investigate the involvement of active site residues in binding the sulfonate substrate. Our studies demonstrate the presence of sulfoacetaldehyde reductase in *Bifidobacteria*, with a possible role in isethionate production as a byproduct of taurine nitrogen assimilation.

## Introduction

Isethionate (Ise) is ubiquitous in the environment, and is also present in mammalian tissues [1]. There is no known pathway for isethionate biosynthesis in animals. Instead, it is thought to originate from metabolism of bile salt-derived taurine by anaerobic gut bacteria [1,2]. Both taurine and isethionate serve as substrates for sulfate- and sulfite-reducing bacteria (SSRB) in the gut, which use the sulfonate-derived sulfite as a terminal electron acceptor (TEA) for anaerobic respiration, producing toxic H_2_S [3,4]. Relatively few taurine-reducing SSRB have been isolated, including the prominent human gut SSRB *Bilophila wadsworthia* [4]. In contrast, isethionate-reducing SSRB appear to be common, and include several *Desulfovibrio* and *Desulfitobacterium* species closely related to gut SSRB [3,5].

**Figure 1:**
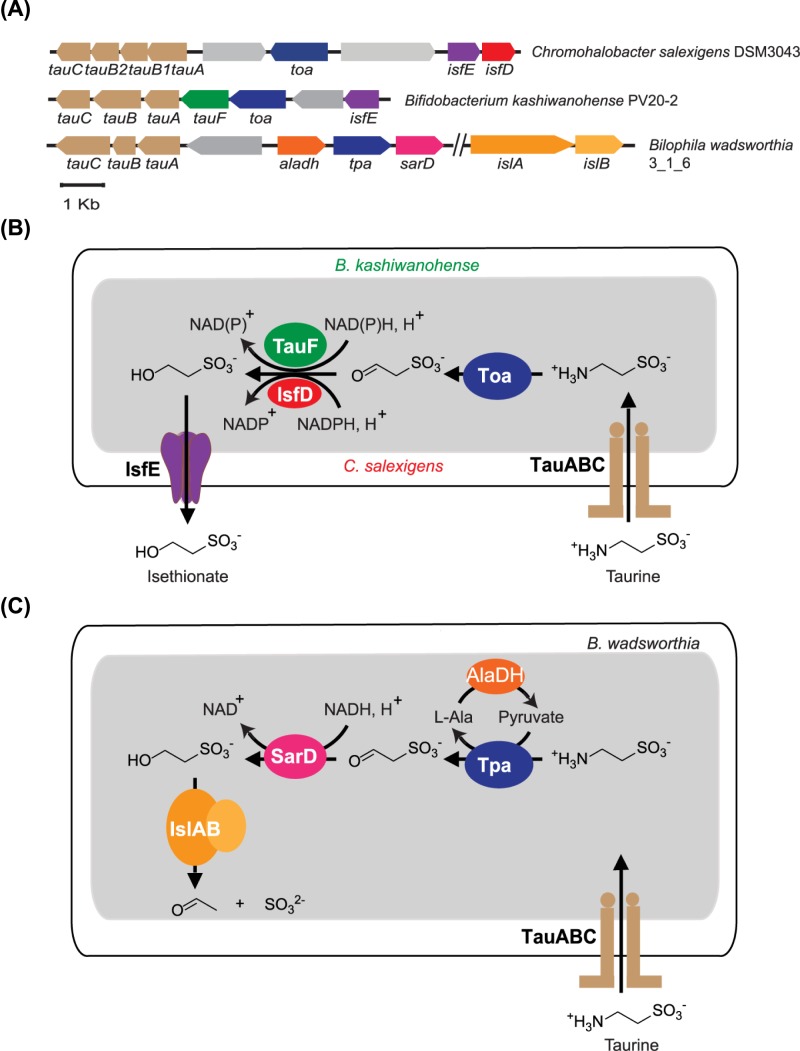
Gene clusters and metabolic pathways involving different sulfoacetaldehyde reductases isozymes (**A**) Gene clusters containing the sulfoacetaldehyde reductases IsfD (in *Chromohalobacter salexigens*), TauF (in *Bifidobacterium kashiwanohense*) and SarD (in *Bilophila wadsworthia*). (**B**) Schematic diagram showing two bacterial taurine nitrogen assimilation pathways relying on different sulfoacetaldehyde reductase isozymes. The pathway in *C. salexigens* relies on IsfD (red), while the putative pathway in *B. kashiwanohense* relies on TauF (green). (**C**) Schematic diagram showing the taurine dissimilation pathway in *B. wadsworthia*.

Conversion of taurine into isethionate has been observed in a mixed culture of anaerobic gut bacteria [1]. Although the specific gut bacterial strains catalyzing this reaction have not been isolated to date, the pathway for isethionate production as a byproduct of taurine nitrogen assimilation has been studied in detail in certain environmental bacteria, including *Klebsiella oxytoca* TauN1 and *Chromohalobacter salexigens* DSM3043, and involves enzymes and transporters arranged in gene clusters (Figure 1A) [6,7]. In this pathway, taurine is imported by a taurine ABC transporter (TauABC), and converted into sulfoacetaldehyde by taurine:oxoglutarate aminotransferase (Toa), generating glutamate as an intermediate for nitrogen metabolism. The NADPH-dependent sulfoacetaldehyde reductase (IsfD), belonging to the SDR family, generates isethionate as a waste produce, which is exported by the putative isethionate exporter (IsfE) (Figure 1B).

We recently reported the crystal structure of IsfD from *K. oxytoca* TauN1, in complex with NADPH and isethionate [8], and showed that it is part of a group of 3-hydroxyacid dehydrogenases, which are ubiquitous in bacteria. However, IsfD-containing gene clusters for isethionate formation have only been predicted in bacteria belonging to the phylum Proteobacteria [6,8], and not in strict anaerobic bacterial taxa that are abundant in the gut. Apart from IsfD, a new sulfoacetaldehyde reductase (SarD), belonging to the metal-dependent alcohol dehydrogenase superfamily (M-ADH) family, was recently reported in *B. wadsworthia* (*Bw*SarD) [9,10]. This enzyme is involved in a pathway for taurine dissimilation, in which isethionate is generated as an intermediate, and further degraded to acetate and H_2_S instead of being secreted (Figure 1C).

While examining the occurrence of taurine-related genes in different human gut bacteria, we noticed the occurrence of a putative gene cluster for taurine nitrogen assimilation, present in *B. kashiwanohense* PV20-2, an isolate from human infant faeces (Figure 1A) [11]. The gene cluster resembles those previously reported by Cook et al. [6]*.* However, instead of IsfD, it contains a member of the M-ADH family, with only 28% identicality to the previously reported *Bw*SarD. Here we report the biochemical and structural characterization of this M-ADH enzyme, which we refer to as *Bk*TauF. We demonstrate that it is a novel sulfoacetaldehyde reductase, which differs from *Bw*SarD in active site residues and metabolic function.

## Materials and methods

### General materials and methods

Lysogeny broth (LB) medium was purchased from Oxoid Limited (Hampshire, U.K.). Methanol and acetonitrile used for liquid chromatography-mass spectrometry (LC-MS) were high-purity solvents from Concord Technology (Minnesota, U.S.A.). Water used in this work was ultrapure deionized water from Millipore Direct-Q. Chemicals were purchased from Sigma–Aldrich, Geneview, J&K, Amresco or Solarbio. Oligonucleotide primers were synthesized by Tsingke Biological Technology (Beijing, China). DNA plasmid Mini prep, fragment gel extraction and restriction endonucleases clean up kits were from Tiangen (Beijing, China). Talon Cobalt resins were purchased from Clonetech (California, U.S.A.). All protein purification chromatographic experiments were performed on an ‘ÄKTA pure’ or ‘ÄKTA prime plus’ FPLC machine equipped with appropriate columns (GE Healthcare). Protein concentrations were determined according to the method of Bradford [12] using bovine serum albumin (BSA) as a standard, or using NanoDrop One (Thermo Fisher Scientific). The extinction coefficient for each protein was obtained using the ExPASy ProtParam tool.

### Gene syntheses and cloning

The codon-optimized gene fragment of *Bk*TauF (UniProt accession: A0A0A7I0A5) was synthesized by General Biosystems (Anhui, China), and inserted into pET-28a-HMT at the *Ssp*I restriction site. The resulting plasmid HMT-*Bk*TauF contains, in tandem: a His_6_-tag, maltose binding protein (MBP) and a Tobacco Etch Virus (TEV) protease cleavage site, followed by *Bk*TauF [13]. The expression of an MBP-*Bk*TauF fusion protein allows purification with an amylose affinity column, followed by cleavage with TEV protease, resulting in high purity of *Bk*TauF.

The original construct was resistant to TEV protease cleavage likely due to structural hindrance. A linker (GGG) between the TEV cleavage site and the N-terminus of *Bk*TauF was thus introduced. New DNA fragment, produced by PCR using the original construct as template and primers HMT-GGG-*Bk*TauF-F and HMT-GGG-*Bk*TauF-R (Supplementary Table S1), was inserted into pET-28a-HMT at the *Ssp*I site by Gibson assembly [14], forming HMT-GGG-*Bk*TauF. Recombinant plasmids were confirmed by DNA sequencing.

### Expression and purification of BkTauF

The HMT-GGG-*Bk*TauF plasmid was transformed into *Escherichia coli* BL21 (DE3) cells. The transformant was grown in LB medium containing kanamycin (50 μg/ml) at 37°C in flasks in a shaker incubator at 220 rpm, and induced for the expression of MBP-*Bk*TauF with 0.3 mM isopropyl β-d-1-thiogalactopyranoside (IPTG) for 16 h at 18°C. Typically, cells from 4 l culture were harvested by centrifugation at 8000×***g*** for 10 min and lysed with French press (Panda plus, Niro Soavi Co., Italy) at 14000 psi in 200 ml buffer A (20 mM Tris/HCl, pH 7.5, 200 mM KCl, and 5 mM β-mercaptoethanol (BME)) containing 25 µg/ml DNaseI and 1 mM phenylmethanesulfonyl fluoride (PMSF). The lysate was then centrifuged at 20000×***g*** for 10 min at 4°C, and the cell debris was discarded.

Nucleic acid was removed by precipitation with 1% streptomycin sulfate. The protein solution was then loaded on to a 10 ml TALON column (GE Healthcare). The column was washed with buffer A, and protein was eluted with buffer A containing 150 mM imidazole. The eluate was dialyzed against buffer A for 4 h to remove imidazole, then loaded on to a column packed with 40 ml amylose resin (New England Biolabs, Massachusetts, U.S.A.). The amylose column was washed with buffer A, and the protein was eluted with buffer A containing 10 mM maltose. TEV protease was added (TEV/*Bk*TauF 1:20 mass ratio), and the solution was dialyzed overnight against 2 l buffer A. The dialyzed sample was loaded on to a 10 ml TALON column to retain His_6_-MBP and His_6_-TEV protease. The flow-through was collected and dialyzed against 2 l of buffer B (20 mM Tris/HCl, pH 8.0, 5 mM BME) for 4 h before it was loaded on to a 10 ml Q sepharose high performance column (GE Healthcare). The column was eluted with a linear salt gradient from 100 to 500 mM KCl. A prominent peak containing *Bk*TauF was collected and concentrated to a final volume of 4 ml (5 mg/ml) using a centrifugal concentrator (30 K MWCO; Millipore). This protein solution was then injected to a Superdex200 gel filtration column and eluted with buffer C (20 mM Tris pH 7.5, 200 mM KCl, 1 mM dithiothreitol (DTT)). The eluate from gel filtration column was re-concentrated and the buffer exchanged by repeated concentration and dilution with storage buffer (10 mM HEPES, pH 7.4, 50 mM KCl, 1 mM TCEP (tris (2-carboxyethyl)phosphine)). The concentrations of purified *Bk*TauF were calculated from its absorption at 280 nm (ԑ_280 nm_ = 22920 M^−1^cm^−1^), measured using a NanoDrop One. The final protein concentration is approximately 10 mg/ml. The purified protein was examined on a 10% SDS polyacrylamide gel.

### Determination of the oligomeric state of BkTauF

A 5 mg/ml solution of *Bk*TauF was analyzed by gel filtration as described above, with a 4 ml injection volume and elution with buffer C at 2 ml min^–1^. A solution of molecular weight markers was analyzed under the same conditions. Thyroglobulin bovine (669 kDa), Horse apoferritin (443 kDa), Sweet potato β-Amylase (200 kDa), BSA (66 kDa), Bovine carbonic anhydrase (29 kDa) were used as molecular weight markers (Sigma MWGF 1000-1KT). The molecular weights of the proteins analyzed by gel filtration were calculated from their elution volume using a second-degree polynomial for the relationship between log (molecular weight) and retention time.

### Crystallization, data collection and structure determination of BkTauF

Initial screening of *Bk*TauF crystals was performed using an automated liquid handling robotic system (Gryphon, Art Robbins, California, U.S.A.) in 96-well format by the sitting-drop vapor diffusion method. The screens were set up at 295 K using various sparse matrix crystal screening kits from Hampton Research and Molecular Dimensions. Several crystallization conditions gave diamond-shaped crystal. After further optimization using the hanging-drop vapor-diffusion method in 24-well plates, we obtained crystals large enough for single crystal X-ray diffraction studies. The best condition yielding crystals was 0.2 M ammonium acetate, 0.1 M Bis-Tris, pH 5.5, 25% W/V PEG 3350. Crystals were flash-cooled in liquid nitrogen using reservoir solution containing 30% glycerol as cryo-protectant. Diffraction data were collected on BL17U1 and BL18U1 at Shanghai Synchrotron Radiation Facility (SSRF). The dataset was indexed, integrated and scaled using HKL3000 suite [15]. Molecular replacement was performed by PHENIX [16] using 1VHD as a search model [17]. The structure was manually built according to the modified experimental electron density using Win Coot [18] and further refined by PHENIX [16] in iterative cycles. Table 1 contains the statistics for data collection and final refinement. All structural figures were generated with UCSF Chimera [19]

**Table 1:** Data collection and refinement statistics for the *Bk*TauF_apo (PDB ID:6JKO) and *Bk*TauF_NAD^+^ crystal (PDB ID:6JKP)

Crystals	TauF_apo	TauF_NAD^+^
λ for data collection (Å)	0.9795	0.9795
**Data collection**		
Space group	P 1 21 1	P 1 21 1
**Cell dimension (Å)**		
a, b, c (Å)	70.468 112.502 93.406	70.881 112.317 93.573
α, β, γ (°)	90 107.335 90	90 107.529 90
Resolution	32.3-1.9 (1.968–1.9)	42.19-3.008 (3.115–3.008)
R_merge_	0.604 (0.082)	0.800 (0.193)
Average I/σ (I)	11.78 (2.0)	6.7 (1.2)
Completeness (%)	99.50 (95.71)	96.92(83.03)
Redundancy	3.5/3.4	2.8/3.4
Z	4	4
**Refinement**		
Resolution	32.3-1.9 Å	42.19-3.008 Å
Number of reflections	108623	27019
R_factor_/R_free_ (10% data)	0.1566/0.1927	0.1998/0.2557
RMSD length (Å)	0.006	0.002
RMSD angle (°)	0.76	0.45
**Number of atoms**		
Protein	1504	1504
Ligands	4	117
Water	1107	1
**Ramachandran plot (%)**		
Most favored	98.66	96.26
Additionally allowed	1.34	3.54

### Sulfoacetaldehyde reduction assays

The substrate sulfoacetaldehyde was introduced as a bisulfite adduct using the method reported by Denger and Cook [20]. To determine the optimal pH for sulfoacetaldehyde reduction, a 200 μl reaction solution containing one of various buffers, pH in a range of 4.0–11.0, 5 mM sulfoacetaldehyde, and 1 mM NADH, was pre-mixed in a 96 well plate, and the reaction was initiated by the addition of 0.5 μg *Bk*TauF. The absorbance at 340 nm was monitored using a Tecan M200 plate reader with 15 s intervals for 2–3 min at room temperature (RT).

To determine the Michaelis–Menten parameters for sulfoacetaldehyde reduction, the reaction was conducted in 50 mM Tris/HCl at the optimal pH of 7.5, and the concentration of one substrate was varied (0–2.3 mM for sulfoacetaldehyde, 0–0.8 mM for NADH/NADPH), in the presence of a saturating concentration of the second substrate (0.5 mM NADH or 5 mM sulfoacetaldehyde). ΔA_340nm_ and the extinction coefficient of NAD(P)H (6220 M^−1^cm^−1^) were used to calculate the rates of the reactions. GraphPad Prism 6.0 was used to extract the kinetic parameters.

### Isethionate oxidation assays

A 200 μl reaction solution containing 50 mM as one of various buffers in a range of pH 8.0–11.5, 0.1 M isethionate and 1 mM NAD^+^, was pre-mixed in a 96-well plate, and the reaction was initiated by the addition of 2 μg *Bk*TauF. The absorbance at 340 nm was monitored using a Tecan M200 plate reader with 15 s intervals for 2–3 min at RT.

To determine the Michaelis–Menten parameters for isethionate oxidation, the reaction was conducted in 50 mM 3-(cyclohexylamino)-2-hydroxy-1-propanesulfonic acid (CAPSO), at the optimal pH of 10.0, and the concentration of one substrate was varied (0–80 mM for isethionate, 0–1 mM for NAD^+^ or 0–5 mM for NADP^+^), in the presence of a saturating concentration of the second substrate (1 mM NAD^+^ or 100 mM isethionate).

To test the substrate specificity of *Bk*TauF, the reaction mixture contained 50 mM CAPSO, pH 10.0, 2 μg *Bk*TauF, 1 mM NAD^+^, and 0.1 M substrate (ethanol (EtOH), ethylene glycol (EG), ethanolamine (EA), 3-hydroxypropane-1-sulfonate, 3-hydroxypropionic acid (3HPA) or isethionate). To compare the activities of wild-type (WT) *Bk*TauF and its mutants, the reaction mixture contained 50 mM CAPSO, pH 10.0, 2 μg of the WT or mutant *Bk*TauF, 0.1 M isethionate and 1 mM NAD^+^.

### LC-MS detection of sulfoacetaldehyde formation

The sulfoacetaldehyde product of isethionate oxidation with *Bk*TauF was detected by derivatization with 2,4-dinitrophenylhydrazine (DNPH) (J&K). A 200 μl reaction mixture, containing 50 mM CAPSO, pH 10.0, 5 μg *Bk*TauF, 0.1 M isethionate and 1 mM NAD^+^, was incubated for 10 min at 30°C. Two negative controls, omitting either *Bk*TauF or isethionate, were also prepared. One hundred microliters of reaction solution was mixed with 1.1 ml of sodium acetate solution (0.73 M), then 800 μl DNPH (0.04%) solution was added. The mixture was incubated at 50°C for 1 h and then filtered prior to LC-MS analysis.

LC-MS analysis was performed on an Agilent 6420 Triple Quadrupole LC/MS instrument (Agilent Technologies), on an Agilent ZORBAX SB-C18 column (4.6 × 250 mm). The column was equilibrated with 75% of 0.1% formic acid in H_2_O, 25% of 0.1% formic acid in CH_3_CN, and developed at a flow rate of 1.0 ml/min from 25 to 65% CH_3_CN. UV detection was set at 360 nm.

### Reconstitution of the metal cofactor for BkTauF

The *Bk*TauF was dialyzed at 4°C overnight against storage buffer containing 10 mM EDTA, and then against storage buffer without EDTA. The metal cofactor was then reconstituted by incubation with a 20-fold excess of (NH_4_)_2_Fe(SO_4_)_2_.6H_2_O or ZnSO_4_ for 30 min at RT. Reconstitution with Fe^2+^ was carried out under an argon atmosphere [21]. The reconstituted proteins were then assayed in a reaction system containing 2 μg *Bk*TauF, 50 mM CAPSO buffer pH 10.0, 0.1 M isethionate and 1 mM NAD^+^, total volume 200 μl in a 96-well plate. The absorbance at 340 nm was monitored using a Tecan M200 plate reader with 15 s intervals for 2–3 min at RT.

### Site-directed mutagenesis

Three single amino acid point mutations in the enzyme active site, F252A, T257A, F265A, were introduced by site-directed mutagenesis using primers listed in Supplementary Table S1, and confirmed by sequencing. A 25 μl of PCR contained 100 ng HMT-GGG-*Bk*TauF plasmid as template, 0.4 μM forward and reverse primers, and the Fast Alteration DNA Polymerase (KM101 from Tiangen, Beijing, China). The 17-cycle PCR mixture was digested by *Dpn*I to remove the template before transformed into *E. coli* competent cells provided by the manufacturer (Tiangen). The WT and mutant *Bk*TauF proteins were purified by Talon affinity column, followed by TEV protease cleavage. The cleaved protein was collected in the flow-through when applied to the same Talon cobalt column to retain His_6_-MBP and His_6_-TEV, and concentrated for enzyme activity assays using isethionate and NAD^+^ as substrates. *Bk*TauF purified with this protocol was approximately 85% pure.

### Sequence alignments

MUSCLE [22] was used to construct multiple sequence alignments. Sequence logos were plotted using WebLogo [23].

## Results

### BkTauF is a dimer in solution

**Figure 2:**
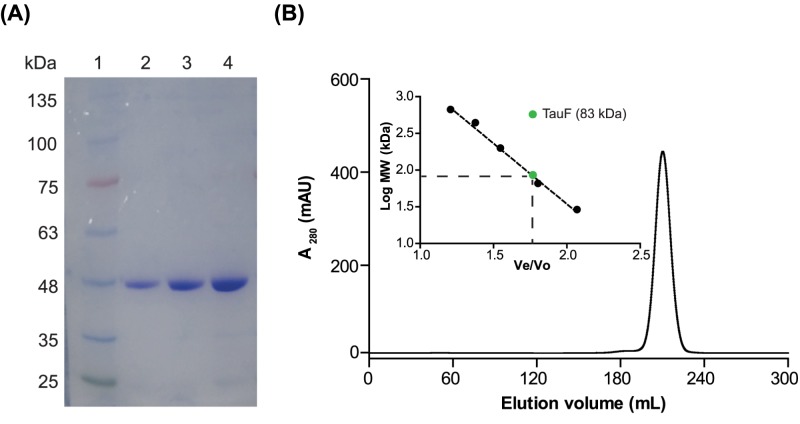
SDS/PAGE and gel filtration analyses of *Bk*TauF (**A**) SDS/PAGE analysis. 10% SDS gel with: lane 1, molecular weight marker; lanes 2–4, 1, 2, 4 µg of *Bk*TauF. (**B**) Elution profile of *Bk*TauF using Superdex 200 gel filtration chromatography to determine its molecular weight, estimated to be 83 kDa.

*Bk*TauF was recombinantly produced and purified to near homogeneity through multiple chromatographic steps. The purified protein was examined on a 10% SDS/PAGE gel (Figure 2A). The gel filtration elution profile of purified *Bk*TauF showed a single symmetric peak centered at 208.6 ml (Figure 2B). The observed molecular weight for *Bk*TauF was 83 kDa (Figure 2B, inset), whereas the calculated molecular weight for *Bk*TauF monomer is 41 kDa. This suggests that *Bk*TauF exists as a dimer in solution.

### Enzyme kinetics of BkTauF

*Bk*TauF catalyzed both sulfoacetaldehyde reduction and isethionate oxidation, as measured using a continuous spectrophotometric assay monitoring the decrease/increase in absorbance at 340 nm due to consumption/production of NAD(P)H. The optimal reaction pH for sulfoacetaldehyde reduction was 7.5, while that for isethionate oxidation was 10.0 (Supplementary Figure S1).

Enzyme kinetic parameters for the forward and reverse reactions were measured at the respective optimal pH conditions and summarized in Table 2. Although both NADH and NADPH could serve as substrates, the *K*_M_ for NADH was ten-fold lower than that of NADPH (Table 2 and Supplementary Figure S2), suggesting a preference for NADH as the reductant. This is in contrast with IsfD, which uses NADPH but not NADH as a reductant [6,8].

**Table 2:** Enzyme kinetic parameters

Substrate	*k_cat_* (s^−1^)	*K*_M_ (mM)	*k_cat_/K*_M_ (M^−1^s^−1^)
Sulfoacetaldehyde	22.83 ± 0.68	0.50 ± 0.04	4.61 × 10^4^
Isethionate	0.97 ± 0.03	4.44 ± 0.59	2.18 × 10^2^
NADH	18.67 ± 0.52	0.06 ± 0.01	3.01 × 10^5^
NADPH	18.99 ± 1.13	0.73 ± 0.08	2.62 × 10^4^
NAD^+^	1.30 ± 0.02	0.08 ± 0.00	1.58 × 10^4^
NADP^+^	0.80 ± 0.02	0.77 ± 0.08	1.03 × 10^3^

### LC-MS detection of oxidation of isethionate to sulfoacetaldehyde by BkTauF

**Figure 3:**
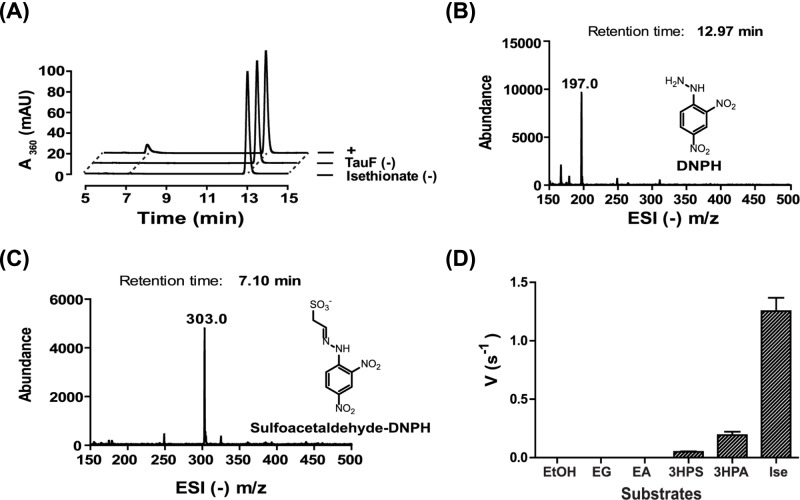
LC-MS assay to detect sulfoacetaldehyde formation and spectroscopic assay to demonstrate substrate specificity (**A**) The elution profile of the LC-MS assays monitoring absorbance at 360 nm. (**B**) The ESI (−) m/z spectrum of the DNPH peak in A). (**C**) The ESI (−) m/z spectrum of the sulfoacetaldehyde-DNPH peak in (A)). (**D**) Spectroscopic assays monitoring absorbance at 340 nm using various alcohol substrates including EtOH, EG, EA, 3-hydroxypropane-1-sulfonate (3HPS), 3HPA and isethionate (Ise).

To demonstrate that sulfoacetaldehyde is the product of isethionate oxidation by *BkTauF*, we carried out LC-MS analysis following derivatization of the product with DNPH [8]. The LC elution profile for the assay mixture contained two major peaks with retention time 7.10 and 12.97 min corresponding to sulfoacetaldehyde-DNPH and DNPH, respectively (Figure 3A). The sulfoacetaldehyde-DNPH peak was absent from the negative controls omitting either *Bk*TauF or isethionate. The ESI (−) (m/z) for both peaks matched with the calculated mass (Figure 3B,C).

### Substrate specificity of BkTauF

Various alcohols were tested as substrates for *Bk*TauF. No activity was detected for EtOH, EG or EA. However, 5 and 15% of enzyme activity were detected for 3-hydroxypropane-1-sulfonate and 3HPA, respectively, possibly reflecting structural similarities to isethionate (Figure 3D).

### The metal cofactor of BkTauF

Enzymes in the M-ADH family are known to utilize Zn^2+^ or Fe^2+^ as the catalytic metal [24]. Catalytic activity of *Bk*TauF was abolished by chelation with EDTA, and recovered up to 85% by addition of Zn^2+^ and 15% by addition of Fe^2+^, suggesting that the physiological metal cofactor for *Bk*TauF is Zn^2+^ (Supplementary Figure S3).

### Crystal structure of BkTauF

**Figure 4:**
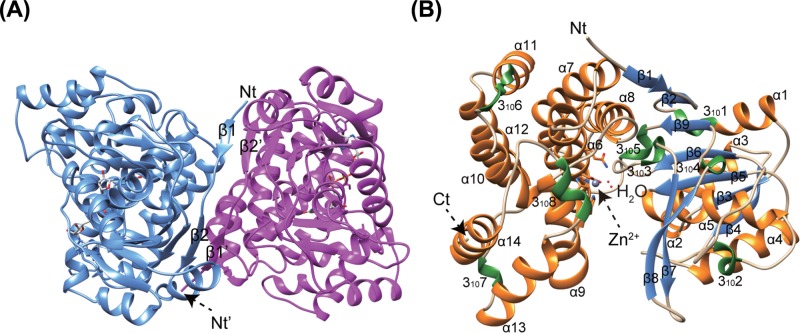
Crystal structures of *Bk*TauF (**A**) Quaternary structure of *Bk*TauF. Strands β1 and β2 involved in dimer interface subunit interaction are labeled. (**B**) Subunit structure of *Bk*TauF is shown in ribbon. α-helices, β strands and 3_10_ helices are shown in orange, blue, and green, respectively.

Crystal structures of *Bk*TauF, both in the apo form and in complex with the NAD^+^ cofactor, were solved at 1.9 and 3.0 Å resolution, respectively. The asymmetric unit contains four monomers. The quaternary prediction server PISA suggests that *Bk*TauF forms a dimer, consistent with its oligomeric state in solution determined by gel filtration chromatography (Figure 2B). The dimerization interface resembles that of *E. coli* lactaldehyde reductase FucO (PDB: 1RRM) [25], stabilized by an anti-parallel β sheet formed between the N-terminal β1 and β2 strands of the two protomers (Figure 4A). Each monomer consists of an N-terminal domain (α1–α5 and β1–β9; residues 1–178) with Rossmann fold, and a C-terminal helical domain (α6-α14; residues 179–375), similar to other structurally characterized M-ADH enzymes (Figure 4B).

**Figure 5:**
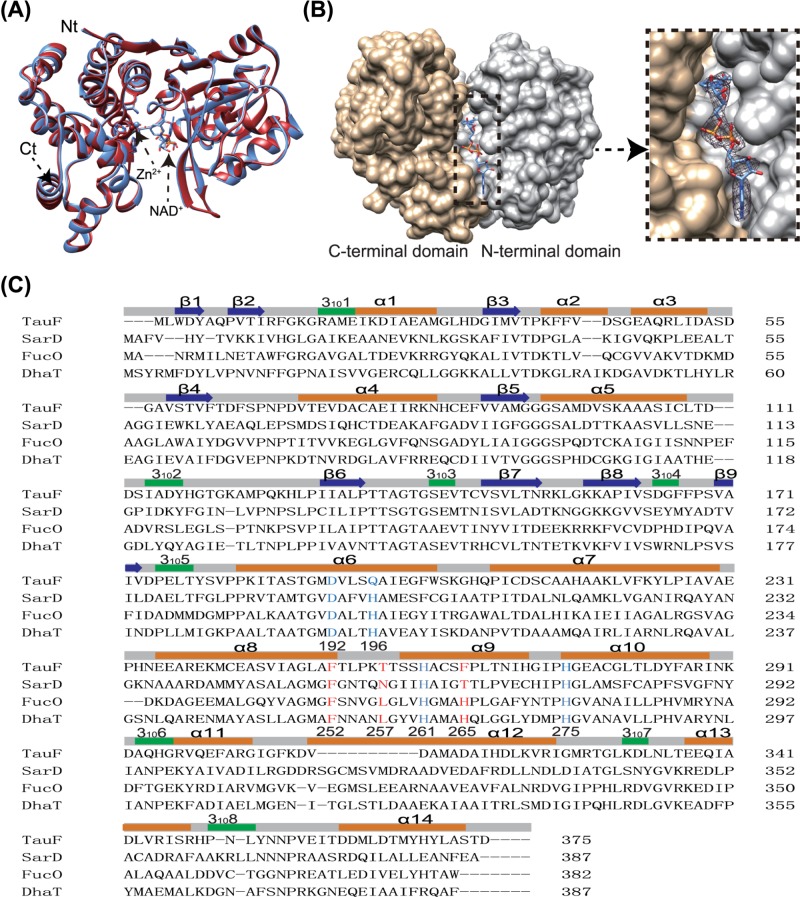
Nucleotide interaction in the structure of *Bk*TauF and structure-based sequence alignments (**A**) The apo and holo (in complex with NAD^+^) structures of *Bk*TauF in red and blue, respectively, are superimposed. (**B**) Surface diagram show the nucleotide binding pocket in *Bk*TauF. The N- and C-terminal domains are displayed in gray and tan, respectively. Inset: zoomed-in view with 2mFo-DFc electron density map of NAD^+^, displayed at 1σ. (**C**) Structure-based sequence alignments (Sequence identities between *Bk*TauF, *Bw*SarD, *Ec*FucO and *Kp*DhaT are 28.1, 28.1 and 30.5%, respectively). The zinc ion coordination residues are highlighted in blue, and the substrate-interacting residues are highlighted in red.

The apo and NAD^+^-bound structures are nearly identical, with RMSD of 0.37 Å over 375 C_α_ atoms (Figure 5A). The cleft between N-terminal domain and C-terminal domain accommodates the protein active site containing NAD^+^ and the catalytic divalent metal ion. The electron density is well defined for NAD^+^ (Figure 5B), which is bound in a configuration similar to that in other M-ADH enzymes [25]. Out of the four monomers in the asymmetric unit, only one contains the catalytic Zn(II) ion, coordinated to Asp^192^, Gln^196^, His^261^ and His^275^. Structure-based sequence alignments between *Bk*TauF, *Bw*SarD, FucO (PDB: 1RRM) [25], and DhaT (PDB: 3BFJ) [26] show conservation in secondary structure, and metal-coordinating residues except that Gln^196^ is atypical in the M-ADH family, with His being more common at that position (Figure 5C).

### Site-directed mutagenesis

Despite attempts to soak the crystals with isethionate, we were unable to obtain a structure with the sulfonate substrate bound. Furthermore, the putative isethionate-binding site adjacent to the catalytic Zn^2+^ is very open, which precluded molecular docking. The present crystal structure could represent an ‘open’ conformation, and further conformational changes could occur subsequent to isethionate binding to form an enclosed active site resembling that of other M-ADH enzymes like FucO [25]. Nevertheless, we attempted to identify candidate active-site residues responsible for interaction with isethionate by examining the crystal structure.

**Figure 6:**
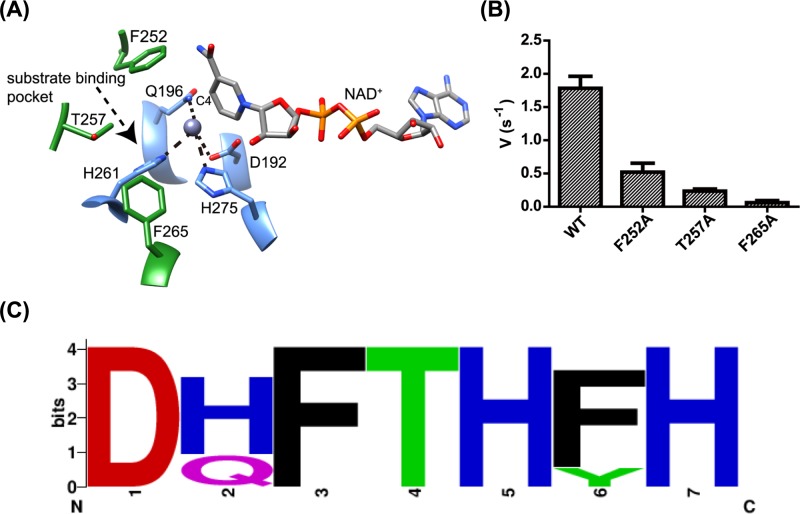
Active site structure and residues of *Bk*TauF (**A**) Zoomed-in view of the active site of *Bk*TauF. The substrate-interacting residues are displayed in green and labeled. Metal-coordinated residues are displayed in blue and labeled. Metal coordination is indicated by dashed lines. (**B**) Enzyme activities of *Bk*TauF active site mutants. (**C**) Sequence logo showing the conservation of active site residues (D192, Q196, F252, T257, H261, F265 and H275) in close homologs of *Bk*TauF in the UniRef cluster UniRef50_ A0A0J8DBY5.

The position of isethionate is constrained by the requirements of the M-ADH catalytic mechanism, which requires coordination of the hydroxyl O-atom to Zn^2+^, and hydride transfer from C_1_ of isethionate to C4 of the NAD^+^. We identified Phe^252^, Thr^257^ and Phe^265^ surrounding the active-site cavity as potential substrate-interacting residues (Figure 6A). Alanine mutants of these three residues were assayed for isethionate oxidation, and their activities were compared with that of the WT enzyme. Activities were almost completely abolished in the T257A and F265A mutants, while the F252A mutant retained approximately 28% of the WT activity (Figure 6B). We examined the 65 *Bk*TauF homologs in the UniRef cluster UniRef50_ A0A0J8DBY5 (where each member shares ≥50% sequence identity and ≥80% overlap with the seed sequence of the cluster [27], and observed that Phe^252^ and Thr^257^ are conserved in these proteins, while Phe^265^ is conservatively replaced with Tyr in a number of proteins (Figure 6C), consistent with their association with the active-site cavity in these proteins. In addition, the Zn^2+^ binding amino acid Gln^196^ is poorly conserved and replaced with the isosteric His in a number of proteins.

### Gene clusters for taurine nitrogen assimilation in anaerobic fermenting bacteria

**Figure 7:**
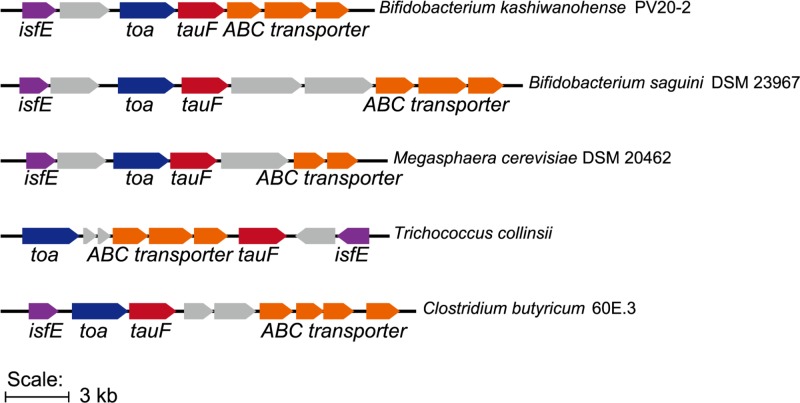
Gene clusters containing close homologs of *Bk*TauF Many of the close homologs of *Bk*TauF in the UniRef cluster UniRef50_ A0A0J8DBY5 are present in strict anaerobic bacteria, and associated with genes putatively involved in taurine nitrogen assimilation and isethionate secretion.

We examined the genome neighborhood of 65 *Bk*TauF homologs in UniRef50_ A0A0J8DBY5, and observed the occurrence of TauF-containing gene clusters similar to that of *B. kashiwanohense*, present in several fermenting bacteria including strict anaerobes from the human gut microbiome (Figure 7). These resemble the IsfD-containing gene clusters for taurine nitrogen assimilation.

## Discussion

Determination of the crystal structure of *Bk*TauF in complex with NAD^+^, together with mutagenesis of active site residues, enabled us to better understand the structural requirements for substrate binding and catalysis. Interestingly, the primary sequence of *Bk*TauF is distantly related to that of the previously reported *Bw*SarD (only 28% identity), and putative substrate-binding active site residues determined for *Bk*TauF are not conserved in *Bw*SarD, suggesting convergent evolution of SarD activity in these two members of the M-ADH family.

The characterization of *Bk*TauF expands the number of bacterial candidates that may be responsible for the conversion of taurine into isethionate in the mammalian body. The previously reported IsfD-dependent pathways are largely found in Proteobacteria, including *Klebsiella pneumoniae*, a facultative anaerobe that is found in low abundance in the human gut [25]. By contrast, close homologs of *Bk*TauF are present largely in strict anaerobic bacteria, including Bifidobacteria and Clostridia, which are highly abundant in the gut. The reason for the different distribution of IsfD and TauF is unclear, but may be related to their different specificities for the nucleotide substrate (NADPH for IsfD and NADH for TauF).

The recently reported taurine dissimilation pathway in *B. wadsworthia* involves SarD-dependent conversion of taurine into isethionate. Cleavage of isethionate by IslA releases sulfite, which is then reduced to H_2_S (Figure 1C) [9,10]. Many SSRB, including the prominent human gut SSRB *Desulfovibrio piger*, lack SarD and only possess the latter half of the pathway for isethionate dissimilation [10]. Further investigation is required to determine whether TauF-dependent conversion of taurine into isethionate in fermenting bacteria could play a role in supplying isethionate to SSRB, thus completing a critical link in gut H_2_S production.

## Supporting information

**Supplementary Figure S1:** 

**Supplementary Figure S2:** 

**Supplementary Figure S3:** 

## Abbreviations

*Bk*TauF: *Bifidobacterium kashiwanohense* TauF
*Bk*Toa: *Bifidobacterium kashiwanohense* Toa
BME: β-mercaptoethanol
BSA: bovine serum albumin
*Bw*SarD: *Bilophila wadsworthia* SarD
CAPSO: 3-(cyclohexylamino)-2-hydroxy-1-propanesulfonic acid
DNPH: 2,4-dinitrophenylhydrazine
EA: ethanolamine
EG: ethylene glycol
EtOH: ethanol
Ise: isethionate
IsfD: NADPH-dependent sulfoacetaldehyde reductase
LB: lysogeny broth
M-ADH: metal-dependent alcohol dehydrogenase superfamily
MBP: maltose binding protein
MUSCLE: multiple sequence comparison by Log-expectation
PDB: protein data bank
RT: room temperature
SSRB: sulfate- and sulfite-reducing bacteria
TEV: tobacco etch virus
WT: wild-type
3HPA: 3-hydroxypropionic acid
