# Supplementary figures and images for: Identification and characterization of a new sulfoacetaldehyde reductase from the human gut bacterium Bifidobacterium kashiwanohense
Source: Biosci Rep. 2019 Jun 20;39(6):BSR20190715. doi: 10.1042/BSR20190715 (PMC6616044; doi:10.1042/BSR20190715)

**A**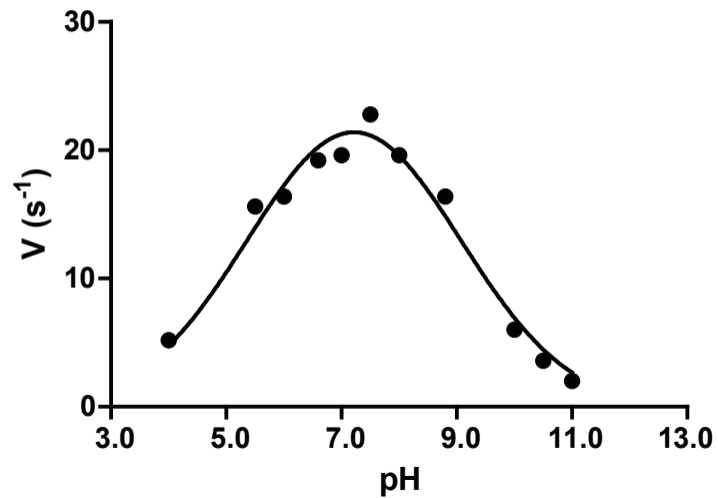**B**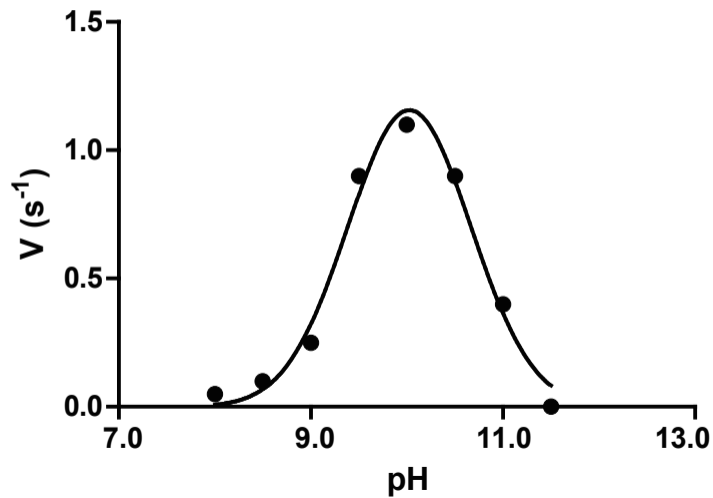

**A**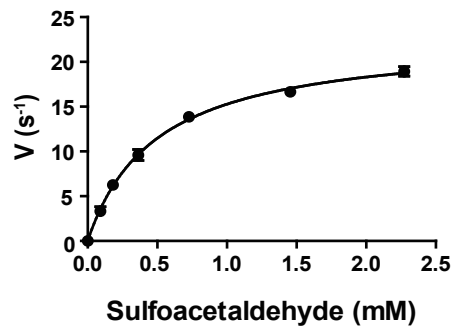**B**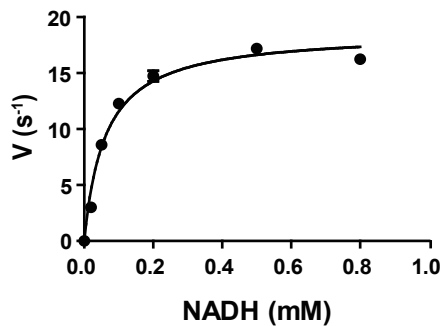**C**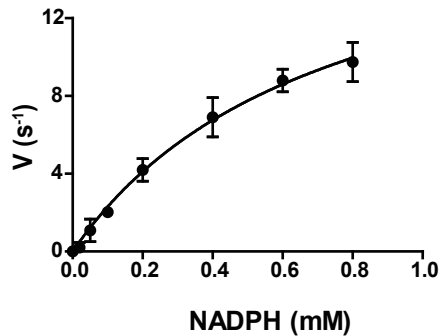**D**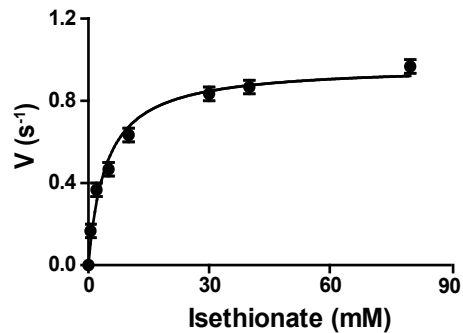**E**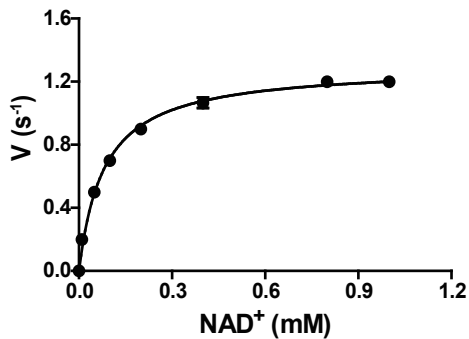**F**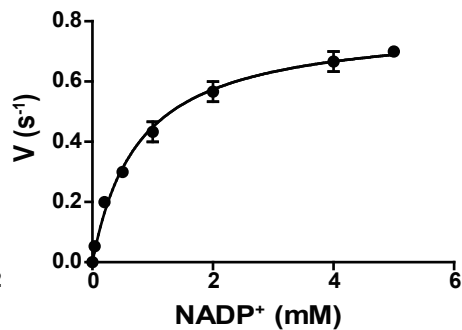

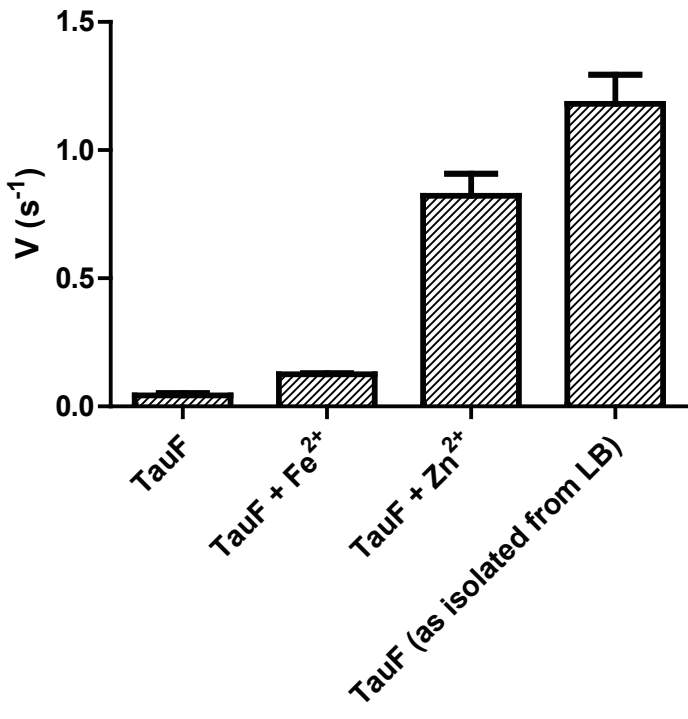

Supplement: Supplementary file 1 [file bsr20190715_Supp1.pdf]
